# A novel multi-item joint replenishment problem considering multiple type discounts

**DOI:** 10.1371/journal.pone.0194738

**Published:** 2018-06-01

**Authors:** Ligang Cui, Yajun Zhang, Jie Deng, Maozeng Xu

**Affiliations:** 1 School of Economics and Management, Chongqing Jiaotong University, Chongqing, 400074, P.R. China; 2 School of Business Administration, Guizhou University of Finance and Economics, Huaxi University Town, Guiyang, 550025, P.R. China; 3 Intellectual Property Institution of Chongqing, Chongqing University of Technology, Chongqing, 400054, P.R. China; Rice University, UNITED STATES

## Abstract

In business replenishment, discount offers of multi-item may either provide different discount schedules with a single discount type, or provide schedules with multiple discount types. The paper investigates the joint effects of multiple discount schemes on the decisions of multi-item joint replenishment. In this paper, a joint replenishment problem (*JRP*) model, considering three discount (all-unit discount, incremental discount, total volume discount) offers simultaneously, is constructed to determine the basic cycle time and joint replenishment frequencies of multi-item. To solve the proposed problem, a heuristic algorithm is proposed to find the optimal solutions and the corresponding total cost of the *JRP* model. Numerical experiment is performed to test the algorithm and the computational results of *JRP*s under different discount combinations show different significance in the replenishment cost reduction.

## 1 Introduction

In the multi-item inventory environment, a joint replenishment policy can generally be defined as the coordination of multiple items that may be ordered jointly from a single supplier [1–3]. Traditionally, two types of ordering cost, the major ordering cost related to ordering times and the minor ordering cost related to each item, in a two-layer supplying system, within which a buyer placing an order to a supplier for a number of different items, are assumed [4]. It is believed that a well planned joint replenishment policy can bring great savings for both buyers and suppliers [2, 5–8]. Henceforth, the joint replenishment problem (*JRP*) has received extensive attention from both practitioners and researchers.

Practically, a buyer is more willing to accept a price break after purchasing a large amount of the supplier’s product, while the motivations for a supplier offering quantity discounts is either to pursue the price discriminate or to reduce the operating cost [9] and control operating risks [10]. For example, several discounts, e.g. percentage-based discount, dollar value discount, and free shipping or free gift on different products are adopted by some B2C e-business to attract more consumers’ buying. In the early stage of the *JRP* research, the benefits obtained by performing joint replenishment policy are solely assumed as the savings in ordering cost through group replenishing different items [1, 4, 11]. However, a performed joint replenishment policy with conventional *JRP* assumptions increases the inventory level and the system cost of the buyer for controlling inventory [2]. While in another aspect, in order to promote the buyer to purchase more items, the supplier usually provides the buyer discount offers to balance the buyers’ inventory level and the inventory carrying cost.

Furthermore, in light of different items show different cost features in manufacturing, supplying and storing, some more flexible discount offers are preferred by the supplier according to the specific supplied items. In reality, comparing to all items that being offered with one discount type, it is very common for suppliers to make a comprehensive decision according to the orders on hand and provide a mixed discount type offer, the reason lies in that multiple discounts can help the supplier make a more flexible selling strategy. Hence, before constructing *JRP* model with multiple discounts, the differences of discounts should be specified.

Thanks to the positive benefits of discounts, various discounting schemes are offered by the suppliers in practice and discussed by researchers. For example, all-unit quantity scheme is a widely utilized scheme as it directly links the ordering prices and quantities of the items and is easy to perform in practice [12]. Incremental quantity discount is another commonly applied discount scheme that the supplier would benefit more as only those ordered unit exceeds certain amount can be offered a lower price. While the total business volume discount scheme is very convenient to apply in the multi-item situation [13, 14]. The motivations for the buyers and suppliers to perform different discount schemes may differ, but it has been testified that the buyer and the supplier would be coordinated if the transfer price or cost is set optimally based on the discounting schemes [15].

The presence of different discount schemes often complicates the item purchasing decisions [16] and sometimes looms the information risks [17, 18]. Thus, in most studies, for ease of processing the discount settings, environments of a single item with multiple discount schemes [13] or multi-item with a single discount scheme [2, 19] are the most welcomed and prevalent research assumptions. However, the research considering both joint replenishment of multi-item and multiple quantity discount scheme offers is rare. Therefore, this study aims to contribute to a supplement research to *JRP* with multiple discount considerations. The main contributions addressed in this research are elaborated as follows.

(1) A new *JRP* model considering three discount types, all-unit quantity, incremental quantity and total business volume, simultaneously is constructed. In practice, a supplier can provide more flexible discount offers in light of particular types of different items. Within this background, the new model is constructed to investigate the joint effects of different discount combinations to the total cost of *JRP*.

(2) An iterative heuristic algorithm is presented to solve the proposed model. In light of the *NP-hard* nature of *JRP*, we design an iterative heuristic algorithm to deal with three quantity discounts sequentially based on two designed solving procedures.

(3) A numerical case is presented to test the effectiveness of the algorithm in solving *JRP*s under different discount combinations. In the numerical experiments, *JRP* with no discount, *JRP* with quantity discount, *JRP* with incremental discount, *JRP* with total business volume discount, and *JRP* with three discounts, are compared and analyzed, respectively.

The rest of this paper is organized as follows. In Section 2, literature on the evolvement of *JRP*s with discount considerations are reviewed. Section 3 presents the assumptions, notations, and the formulation process of the model, the corresponding solving procedures are also given. In Section 4, a *JRP* case is presented as a numerical example to test effects of *JRP*s with different discounts. Section 5, conclusions and focus for future research are provided.

## 2 Literature review

Numerous researchers have made contributions in researching *JRP*s since *JRP* was presented in 1970s. Currently, JRP has already become one of the most important research branch that deal with multi-items. In this part, we limit our focus on the researches of multi-items replenishment with can provide us a clear understanding of the *JRP* models and the solving methodologies in discount environment.

### 2.1 Item replenishment with discount considerations

Item replenishment with discount considerations is a common practice in commercial purchasing activities, however, it is always a great challenge in making a decision on replenishing multi-item with different discount cobinations. In general, item replenishment involves numerous processes and activities, such as demand prediction, supplier selection, price negotiation, and so on [20–22]. The offered discounted prices for the buyer making the replenishment decision becomes even more complicated [16]. Thus, the vast majorities of researchers construct mathematical models to study item replenishment with discount considerations to investigate the connections of the ordering quantities and the ordering cost. Basically, based on the types of items with discount offers, the researches can be classified as the single item replenishment problem and the multi-items replenishment problem.

The single item replenishment problem with discount consideration often reduces to the problem of multi-supplier selection. Within this circumstance, Xia and Wu [16] once noted, no one supplier can fulfill the whole order so that the order is divided from one supplier to multiple suppliers. Thus, multiple sources of items and their extensions are generally considered in many researches, but each supplier is generally assumed to supply a single type of item. For example, Yang et al. [23] focused on obtaining the satisfied replenishment policy to minimize the transportation time and inventory cost in a multi-supplier multi-retailer supply chain, where the transportation cost are discounted according to the ordering quantities of different items. Zhang and Chen [21] constructed a mixed integer programming model to allocate the discounted ordering quantities of a single type of item to multiple suppliers, the objective of the model is to minimize the total cost, including the selecting cost, the procurement cost, the holding cost and the shortage cost. On deciding the purchasing prices of single items, Lee et al. [24] assumed that both all-unit quantity discounts and incremental discounts were provided by parts of suppliers, respectively.

The multi-item replenishment models with discount consideration are usually constructed under the assumption that a supplier fulfills the whole order. Haksever and Moussourakis [25] presented a mixed integer programming model to determine the best-found order quantities of multi-item with incremental quantity discount offered by multiple suppliers. Zhang [26] examined a multi-item newsboy problem and formulated a mixed integer model to investigate the impact of quantity discount and budget constraint to the optimal ordering quantity. Considering the multi-suppliers with the all-unit quantity discount, Shi and Zhang [27] formulated a model to determine the best selling prices and ordering quantities of multi-items simultaneously. Manerba and Mansini [28] made a further extension to the single supplier selection problem and assumed the orders can be fulfilled among different suppliers with the total quantity discount (*TQD*). Based on the work of these forerunners, our research would contribute the literature on investigating the multi-item jointly replenishment problem with multiple discounts.

A general summary of pertinent papers is provided in Table 1.

**Table 1:** Summary of pertinent papers.

Article	Discount	Item	Buyer	Supplier	Model and Solution Algorithm
[16]	All unit discounts	Multi-item	–	Multi-supplier	Multi-objective programming, optimization tool box of Matlab
[23]	All unit discount	Single item	Multi-retailer	Multi-supplier	MIP*, Genetic Algorithm
[21]	All unit discount	Single item	–	Multi-supplier	MIP, Bender’s decomposition heuristic
[24]	All-unit and incremental discounts	Single item	–	Multi-supplier	MIP, Genetic Algorithm
[25]	Incremental quantity discount	Multi-item	A warehouse	–	MIP, multiple software packages
[26]	All unit discount	Multi-item	A newsboy	–	MIP, lagrangian relaxation
[27]	All unit discount	Multi-item	A retailer	Multi-supplier	MIP, lagrangian relaxation
[28]	Total quantity discount	Multi-item	–	Multi-supplier	MIP, a branch-and-cut approach
[29]	Total quantity discount	Multi-item	A buyer	Multi-supplier	MIP, a heuristic algorithm
[30]	All-unit discount	Multi-lane	A distributor	Multi-carrier	MIP, a tabu search algorithm
[2]	All unit discount	Multi-item	A buyer	A supplier	MIP, heuristic algorithms
[13]	All unit discounts, incremental and total volume discounts	Single item	A buyer	Multi-supplier	MIP, a scatter search algorithm
[31]	All unit and incremental discounts	Single item	A centralized buyer	Multi-vendor	Integer lot-sizing model and heuristic algorithms

* MIP is the abbreviation of Mixed Integer Programming.

From Table 1, we observe that (1) a large share of papers are focused on supplier selection problem and supplying assignment problem, only a small number of research papers consider the multi-item joint replenishment problem. (2) three typical discount schemes, all-unit quantity discount, incremental discount and total volume discount, are the most favorite discount structures in the model constructions, but the papers considering multiple discounts are rare. (3) the mixed integer programming (MIP) models are constructed in most papers, but their solution algorithms are different. Therefore, in light of above researches in item replenishment modeling without mixed discount type considerations, our research would provide supplement literature on joint replenishment problem with multiple discounts.

### 2.2 *JRP*s with discount schemes

Since Shu [11] presented *JRP*, *JRP*s have drawn worldwide researchers’ attention. Khouja and Goyal [1] reviewed several extension of *JRP*s, including *JRP* under stochastic [32] and *JRP* under dynamical demand [33]. Other extensions, such as all-unit quantity discount [19], *JRP* under continuous unit cost decrease *JRP* with supplying capacity constraints [34], *JRP* with delivery [35], *JRP* with imperfect items [2] and so on, are developed. Of all *JRP* extensions, one extension of *JRP*, *JRP* with multiple quantity discount schemes, has not been fully considered, though multiple discount combinations are practiced by the practitioners. In general, two strategies, the direct grouping strategy (*DGS*) and the indirect grouping strategy (*IGS*) are raised for grouping items [1]. However, before *DGS* is performed, a predetermined number of groups should be provided under the minimized total cost [36]. Under *IGS*, the replenishment cycle of each item is an integer multiplier of the basic cycle time. The problem is simplified as to determine the basic cycle time and the replenishment frequencies of all items simultaneously. Thus, *IGS* is adopted in the following analysis.

In traditional *JRP*, the ordering quantities are assumed deterministic [37], in which the superiorities of joint replenishment are reflected in but not limited to acquire the savings of ordering cost by group purchasing multi-items. By introducing the all-unit quantity discount to *JRP*, Cha and Moon [19] constructed a *JRP* model with quantity discounts and an efficient heuristic algorithm was developed to solve the proposed model. Moon et al. [38] transformed the single supplier *JRP* with all-unit quantity discount to a multi-supplier and each item is assumed to be purchased from one supplier. Paul et al. [2] formulated a *JRP* model considering the imperfect items and all-unit quantity discount. However, there are no researches considering the mixed quantity discount scheme in *JRP*.

**Fig 1:**
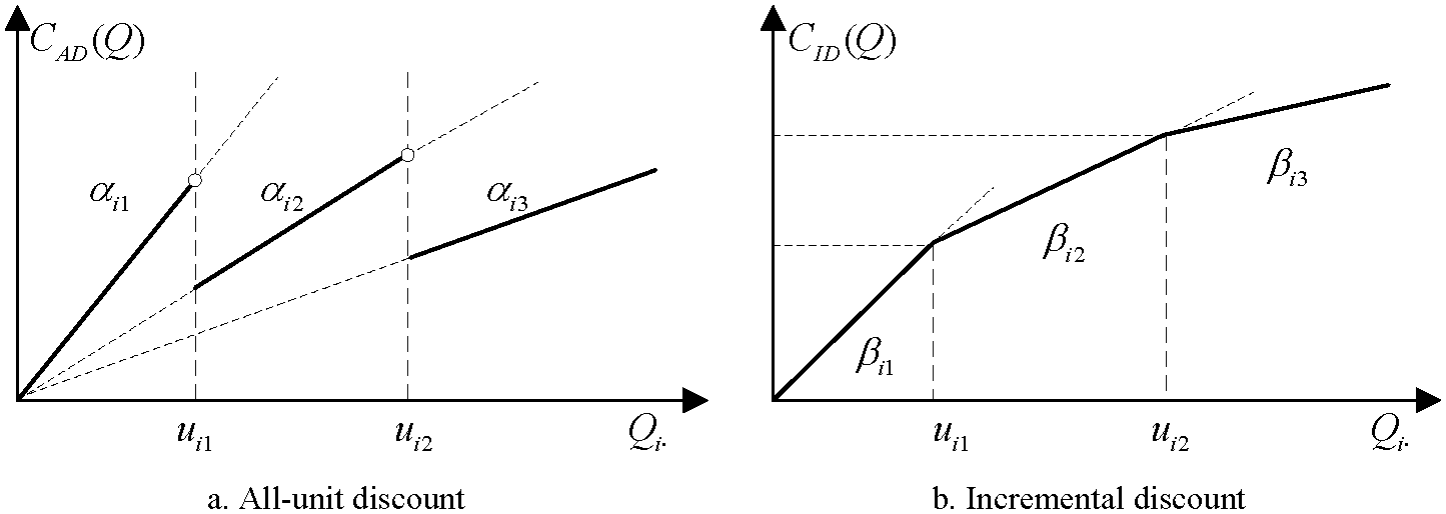
Graphical illustration of two discounts.

When talking about the discount structures, Munson and Rosenblatt [9] pointed that the form of discount may be either all-units or incremental. Three common discount schemes, the all-unit quantity discount, the incremental quantity discount and the total volume discount are commonly applied in model constructions. According to the definition from Lee et al. [24], under the all-unit quantity discount, if the ordering quantity belongs to a specified quantity level predetermined by the supplier, the discounted price is applied to all-units starting from the first unit, see Fig 1a. The incremental quantity discount shows the only difference in that the discounted price of incremental quantity discount is applied to the units inside two continuous quantity breaks, see Fig 1b. While the total business volume discount (*TBD*) scheme or *TQD* presented in Ebrahim et al. [13], Manerba and Mansini [28] and Xia and Wu [16] to illustrate the fluctuation of total business values over the total ordering quantities of all items, which means that a *TBD* represents item aggregation where the price breakpoints are based on the total dollar volume of business over all items ordered from the supplier [9]. Therefore, *TBQ* can be considered as the variation of all-unit or incremental discount. A graphical illustration of the two (all-unit and incremental) discounts is presented in Fig 1.

## 3 Description of the proposed model and the solving algorithm

### 3.1 Problem description, assumptions and notations

In the proposed model, a two-layer supply chain with a supplier (e.g. an item manufacture) and a buyer (e.g. a distribution center or a retailer) is considered. At the supplying side, besides the items supplied with no discount, the supplier also offers three discounts, all-unit quantity discount, incremental discount, and total business volume discount, to the buyer according to the stored items. Moreover, each kind of item can only have one discount type. At the buying side, four types of cost, the major ordering cost, the minor ordering cost, the inventory holding cost, and the item purchasing cost, are considered during the replenishment process. The aim is to find the optimal combination of the basic cycle time and the ordering frequencies of all items with the context of multiple discounts.

The assumptions of the general *JRP* are inherited from the assumptions of the economic ordering quantity (*EOQ*) problem. For example, the demand is assumed to be deterministic and conforms to a uniform distribution, no shortages are allowed, no quantity discount, the holding cost is linear [1], and so on. Based on these assumptions, the assumptions considered throughout this paper are given below:

The demand of each item is deterministic and constant.
No shortages are allowed.
The items are replenished when the inventory level drops to zero.
The inventory holding cost is known and constant.
The order is delivered instantly without the lead-time consumption.
Three discount offered by the supplier.
The discount structures are offered by the supplier and known by buyer.
Each type of items is offered one and only one possible discount scheme

Accordingly, the vectorial sets, indices, and decision variables are given as follows:

*i*: the index of items, and set I={i|i=1,2,⋯,n},
*j*: the index of discount intervals, and set $J=\{j|j=1,2,\cdots ,J_{i}\},$
*n*_0_: the number of items that are offered no discount (ND) by the supplier, and set $N_{0}$ means the items with ND in $N_{0},$
*n*_1_: the number of items that are offered all-unit quantity discount (AD) by the supplier, and set $N_{1}$ means the items with AD in $N_{1},$
*n*_2_: the number of items that are offered incremental discount (ID) by the supplier, and set $N_{2}$ means the items with ID in $N_{2},$
*n*_3_: the number of items that are offered total business volume discount (BD) by the supplier, and set $N_{3}$ means the items with BD in $N_{3},$
*c*_*i*_: unit cost/price of item *i* that the buyer pays to the supplier with ND,
*α*_*ij*_: discounted unit price of item *i* in the *j*-th interval under the AD scheme,
*β*_*ij*_: discounted unit price of item *i* in the *j*-th interval under the ID scheme,
*γ*_*ij*_: discounted rate of item *i* in the *j*-th interval under the BD scheme,
*x*_*ij*_: binary variable: if and only if the order quantity of item *i* falls on the interval of *j*, *x*_*ij*_ = 1, otherwise *x*_*ij*_ = 0,
*μ*_*ij*_: threshold (breakpoint) of each discount interval, and to item $i,0=\mu_{i,0}<\mu_{i,1}<\cdots <\mu_{i,J_{i}}<\mu_{i,J_{i}+1}\leq \infty ,$
*TC*: total annual cost of all items,
*S*: major ordering cost of each order,
*s*_*i*_: minor ordering cost of each item,
*D*_*i*_: demand rate of item *i*,
*h*_*i*_: annual holding cost of item *i*,
*T*: basic cycle time (decision variable), and
*k*_*i*_: integer multiplier of item *i* (decision variable), *k*_*i*_ ∈ *K*.

### 3.2 Model formulation

#### 3.2.1 The general *JRP* model

Under the indirect grouping strategy (*IGS*) [1], *T*_*i*_ for each item *i* is an integer multiple *k*_*i*_ of T. Thus, the replenishment cycle of item *i* is:

$$\begin{array}{c} T_{i}=k_{i}T \end{array}$$ (1)

and the order quantity *Q*_*i*_ of item *i* is:

$$\begin{array}{c} Q_{i}=T_{i}D_{i}=D_{i}k_{i}T \end{array}$$ (2)

The annual total holding cost per unit time is:

$$\begin{array}{c} C_{h}=\sum_{i=1}^{n}Q_{i}h_{i}/2=\frac{T}{2}\sum_{i=1}^{n}k_{i}D_{i}h_{i} \end{array}$$ (3)

And the annual total ordering cost per unit time is:

$$\begin{array}{c} C_{o}=S/T+\sum_{i=1}^{n}\left(s_{i}/\left(k_{i}T\right)\right)=\frac{1}{T}\left(S+\sum_{i=1}^{n}\left(s_{i}/k_{i}\right)\right) \end{array}$$ (4)

Accordingly, the annual total cost per unit time is:

$$\begin{array}{c} TC_{0}\left(T,K\right)=C_{h}+C_{o}=\frac{T}{2}\sum_{i=1}^{n}k_{i}D_{i}h_{i}+\frac{1}{T}\left(S+\sum_{i=1}^{n}\left(s_{i}/k_{i}\right)\right) \end{array}$$ (5)

where *k*_*i*_ ∈ *K*, *i* = 1, 2, ⋯, *n*, and *K* is a set of integer multipliers. Here we call the annual total cost per unit time as the total cost TC, and the objection is to find the minimized *TC* of *JRP*. For a fixed $K=\left(k_{1},\cdots ,k_{n}\right)\in N^{n}$ the optimal value of T* is given by Eq (6) below:

$$\begin{array}{c} T^{*}=\sqrt{2\left(S+\sum_{i=1}^{n}\left(s_{i}/k_{i}\right)\right)/\sum_{i=1}^{n}k_{i}D_{i}h_{i}} \end{array}$$ (6)

Thus, the optimal *TC* is obtained after *T* and *k*_*i*_s have been fixed. The *k*_*i*_ is obtained by referring to the optimal condition presented by Goyal [4], such that

$$\begin{array}{c} k_{i}\left(k_{i}-1\right)\leq \frac{2s_{i}}{D_{i}h_{i}T^{2}}\leq k_{i}\left(k_{i}+1\right) \end{array}$$ (7)

In general, the purchasing cost of items is not included in the total cost of joint replenishment process. In practice, however, most of the practitioners prefer to perform the joint replenishment strategy not only for the sake of acquiring benefits in ordering cost decreasing, but also eager to save more cost through ordering different items in large batches with different of discount offers. Therefore, the total joint cost of *JRP* with no item discount is presented as

$$\begin{array}{c} TC\left(T,K\right)=C_{h}+C_{o}+C_{p}=\frac{T}{2}\sum_{i=1}^{n}k_{i}D_{i}h_{i}+\frac{1}{T}\left(S+\sum_{i=1}^{n}\left(s_{i}/k_{i}\right)\right)+\frac{1}{T}\left(\sum_{i=1}^{n}c_{i}Q_{i}\right) \end{array}$$ (8)

where *C*_*p*_ is the total purchasing cost (we can also call it the *occupational cost* or *inventory carrying cost* per unit time) of items in each order for the buyer, *n* = *n*_0_ + *n*_1_ + *n*_2_ + *n*_3_, and *Q*_*i*_ can be substituted by *D*_*i*_ *k*_*i*_ *T*.

#### 3.2.2 *JRP* with multiple discounts

The total purchasing cost of the buyer depends on the cost structure offered by the supplier. In the following the structures of three mentioned discounts are presented. Here below the cost function of each discount structure is given as

(1) All-unit quantity discount

In the all-unit quantity discount scheme, the supplier offers price discount according to the possible order quantities of different items. The price is stepped down as the ordering quantity of an item increases progressively in different intervals, and the ordering quantity intervals are divided according to the maximum and the minimum ordering data in the supplier’s supplying history. Thus, the total purchasing cost per unit time with all-unit discount is formulated as:

$$\begin{array}{c} C_{AD}=\frac{1}{T}\sum_{i=1}^{n_{1}}\sum_{j\in J}\alpha_{ij}x_{ij}Q_{ij} \end{array}$$ (9)

where $\sum_{j=1}^{J_{i}+1}x_{ij}Q_{ij}=Q_{i}$ and $\sum_{j=1}^{J_{i}+1}x_{ij}=1$, which means that for item *i* for *j* ∈ *J*, *Q*_*ij*_ = *Q*_*i*_ if and only if *μ*_*i*,*j*−1_ ≤ *Q*_*i*_ < *μ*_*i*,*j*_. It is also assumed that the unit price is stepped down as *α*_*i*1_ > *α*_*i*2_ > ⋯ > *α*_*iJ*_*i*__ for item *i*, Fig 1a gives a simple illustration of the all-unit discount. Therefore, if *j* is fixed, the *C*_*AD*_ can be simplified as $C_{AD}=\sum_{i=1}^{n_{1}}\frac{1}{T}Q_{i}\sum_{j\in J}\alpha_{ij}x_{ij}=\sum_{i=1}^{n_{1}}\sum_{j\in J}\alpha_{ij}x_{ij}k_{i}D_{i}$.

(2) Incremental discount

For the incremental discount scheme, the slightly difference comparing to the all-unit quantity discount lies in that the incremental discount applies only when quantity exceeds the price break quantity. The cost function *C*_*ID*_ under incremental discount scheme is given as:

$$\begin{array}{c} C_{ID}=\frac{1}{T}\sum_{i=1}^{n_{2}}\sum_{j\in J}\left(\beta_{ij}\left(Q_{ij}-x_{ij}\mu_{i,j-1}\right)+x_{ij}\sum_{g=1}^{j-1}\beta_{ig}\left(\mu_{i,g}-\mu_{i,g-1}\right)\right) \end{array}$$ (10)

where ∑_*j* ∈ *J*_ *x*_*ij*_ = 1, and if and only if *μ*_*i*,*j*−1_ ≤ *Q*_*ij*_ < *μ*_*i*,*j*_, *x*_*ij*_ equals to 1 and the others equal to 0 for *j* ∈ *J*. It is also assumed that the unit price in this scheme is stepped down as *β*_*i*1_ > *β*_*i*2_ > ⋯ > *β*_*iJ*_*i*__ for item *i*, Fig 1b gives a simple illustration of the incremental discount.

(3) Total business volume discount

In the total business volume discount scheme, supplier offers discount rate according to the total business value of the ordered items, but not to the ordering quantities, and the discount rate breaks are a function of total business volume discount. The structure of total business volume discount has been testified similarly to that in all-unit discount scheme by [13] for single item purchasing. Following the model construction principal for total business volume discount in [14] and [16], the total purchasing cost function *C*_*BD*_ per unit time with total business volume discount is modeled as:

$$\begin{array}{c} C_{BD}=\frac{1}{T}\sum_{i=1}^{n_{3}}\sum_{j\in J}\left(1-\gamma_{ij}\right)x_{ij}c_{i}Q_{i} \end{array}$$ (11)

where $\sum_{j=1}^{J_{i}+1}x_{ij}=1$ and if and only if *μ*_*i*,*j*−1_ ≤ *c*_*i*_ *Q*_*i*_ < *μ*_*i*,*j*_, *x*_*ij*_ = 1, otherwise, *x*_*ij*_ = 0 for *j* ∈ *J*. In this case, there is a need to calculate the total cost of the order firstly before the total business volume discount scheme takes effect. Then, by examining which discount interval the total cost lies in, the price (discount rate) offer is decided. It is also assumed that the unit discount rate is stepped down as *γ*_*i*1_ > *γ*_*i*2_ > ⋯ > *γ*_*iJ*_*i*__. Similarly, if *j* is fixed, the *C*_*BD*_ can also be simplified as $C_{BD}=\sum_{i=1}^{n_{1}}\frac{1}{T}c_{i}Q_{i}\sum_{j\in J}\left(1-\gamma_{ij}\right)x_{ij}=\sum_{i=1}^{n_{1}}\sum_{j\in J}\left(1-\gamma_{ij}\right)x_{ij}c_{i}k_{i}D_{i}$

After three cost functions have been formulated, the total joint cost of *JRP* with multiple discounts is given below:

$$\begin{array}{c} TC^{\prime }\left(T,K\right)=C_{h}+C_{o}+C_{p}^{\prime } \end{array}$$ (12)

where $C_{p}^{\prime }$ is the total item purchasing cost, including the total cost of items purchased with no discount, the all-unit quantity discount, the incremental discount and the total business volume discount, and $C_{p}^{\prime }$ is modeled as

$$\begin{array}{ccc} C_{p}^{\prime } & = & {C_{p}}_{\left(n=n_{0}\right)}+{C_{AD}}_{\left(n=n_{1}\right)}+{C_{ID}}_{\left(n=n_{2}\right)}+{C_{BD}}_{\left(n=n_{3}\right)}\\ & = & \frac{1}{T}(\sum_{i=1}^{n_{0}}c_{i}Q_{i}+\sum_{i=1}^{n_{1}}\sum_{j\in J}x_{ij}\alpha_{ij}Q_{ij}+\sum_{i=1}^{n_{2}}\sum_{j\in J}(\beta_{ij}\left(Q_{ij}-x_{ij}\mu_{i,j-1}\right)+\\ & & x_{ij}\sum_{g=1}^{j-1}\beta_{ig}\left(\mu_{i,g}-\mu_{i,g-1}\right))+\sum_{i=1}^{n_{3}}\sum_{j\in J}\left(1-\gamma_{ij}\right)x_{ij}c_{ij}Q_{ij}) \end{array}$$ (13)

where $\sum_{j=1}^{J_{i}}x_{ij}=1$.

#### 3.2.3 Solutions for *JRP* with multiple discounts

In order to obtain the optimal combination of *T* and *k*_*i*_s that minimizes *TC*′, two remarks below are presented to illustrate the solving process of the proposed model. *JRP* has been testified as the *NP-hard* problem [33], the most effective and efficient methodologies for *JRP*s are the heuristic algorithms. Henceforth, a simple heuristic algorithm is presented in the following contents.

For a given set of *k*_*i*_s, taking the derivative of *TC*′(*T*, *K*) with respect to *T* and let it equal to 0, we have

$$\begin{array}{cc} \frac{\partial TC^{\prime }\left(T,K\right)}{\partial T} & =\frac{1}{2}\sum_{i=1}^{n}k_{i}D_{i}h_{i}-\frac{1}{T^{2}}\left(S+\sum_{i=1}^{n}\left(s_{i}/k_{i}\right)\right)+\frac{\partial C_{p}^{\prime }}{\partial T} \end{array}$$ (14)

while $\frac{\partial C_{p}^{\prime }}{\partial T}$ can be decomposed as $\frac{\partial C_{p}^{\prime }}{\partial T}=\frac{\partial C_{p}}{\partial T}+\frac{\partial C_{AD}}{\partial T}+\frac{\partial C_{ID}}{\partial T}+\frac{\partial C_{BD}}{\partial T}$. As $\frac{\partial C_{p}}{\partial T}=\frac{\partial C_{AD}}{\partial T}=\frac{\partial C_{BD}}{\partial T}=0$, *Q*_*ij*_ = *Q*_*i*_ for a fixed *j*, taking the derivative of *C*_*ID*_ with respect to *T* considering *x*_*ij*_ = 1, we can obtain

$$\begin{array}{ccc} \frac{\partial C_{ID}}{\partial T} & = & \frac{\partial (\frac{1}{T}\sum_{i=1}^{n_{2}}\sum_{j\in J}\left(\beta_{ij}\left(Q_{i}-x_{ij}\mu_{i,j-1}\right)+x_{ij}\sum_{g=1}^{j-1}\beta_{ig}\left(\mu_{i,g}-\mu_{i,g-1}\right)\right))}{\partial T}\\ & = & \frac{\partial \frac{1}{T}\left(\sum_{i=1}^{n_{2}}\sum_{j\in J}\beta_{ij}Q_{i}\right)}{\partial T}+\\ & & \frac{\partial (\frac{1}{T}\sum_{i=1}^{n_{2}}\left(-\beta_{ij}\mu_{i,j-1}+\sum_{g=1}^{j-1}\beta_{ig}\left(\mu_{i,g}-\mu_{i,g-1}\right)\right))}{\partial T}\\ & = & \frac{\sum_{i=1}^{n_{2}}\left(\beta_{ij}\mu_{i,j-1}-\sum_{g=1}^{j-1}\beta_{ig}\left(\mu_{i,g}-\mu_{i,g-1}\right)\right)}{T^{2}} \end{array}$$ (15)

Hence, if we define $\frac{\Delta }{T^{2}}=\frac{\partial C_{ID}}{\partial T}$, we have $\frac{\partial C_{p}^{\prime }}{\partial T}=\frac{\Delta }{T^{2}}$. Δ can also be expressed as $\Delta =\sum_{i=1}^{n_{2}}\left(\beta_{ij}\mu_{i,j-1}-\sum_{g=1}^{j-1}\beta_{ig}\left(\mu_{i,g}-\mu_{i,g-1}\right)\right)$ at the premise that the best purchasing interval of item *i* is ascertained and *x*_*ij*_ = 1. Through decomposition, Δ can be rewritten as

$$\begin{array}{ccc} \Delta & = & \sum_{i=1}^{n_{2}}\left(\beta_{ij}\mu_{i,j-1}-\beta_{i,j-1}\left(\mu_{i,j-1}-\mu_{i,j-2}\right)-\sum_{g=1}^{j-2}\beta_{ig}\left(\mu_{i,g}-\mu_{i,g-1}\right)\right)\\ & = & \sum_{i=1}^{n_{2}}\sum_{g=2}^{j}\left(\left(\beta_{i,g}-\beta_{i,g-1}\right)\mu_{i,g-1}+\beta_{i1}\mu_{i,0}\right) \end{array}$$ (16)

Since *μ*_*i*,0_ = 0 and *β*_*i*,*j*−1_ > *β*_*i*,*j*_, we have Δ < 0 and $\frac{\partial C_{p}^{\prime }}{\partial T}<0$. Consequently, if $S+\sum_{i=1}^{n}\left(s_{i}/k_{i}\right)+\Delta \geq 0$, by solving Eq (14), the optimal *T* (denoted by $\bar{T}$) can be expressed as

$$\begin{array}{c} {\bar{T}}^{=}\sqrt{2(S+\sum_{i=1}^{n}\left(s_{i}/k_{i}\right)+\Delta )/\sum_{i=1}^{n}k_{i}D_{i}h_{i}} \end{array}$$ (17)

where $S+\sum_{i=1}^{n}\left(s_{i}/k_{i}\right)+\Delta \geq 0$, from which we can also obtain that $-(S+\sum_{i=1}^{n}\left(s_{i}/k_{i}\right))\leq \Delta <0$. The next problem is to find feasible Δs. Hence, taking a two-item case for example, the data of the case are provided in Table 2. For each item, the values of $\Delta_{i,j}=\sum_{j=2}^{J_{i}}\left(\left(\beta_{i,j}-\beta_{i,j-1}\right)\mu_{i,j-1}+\beta_{i1}\mu_{i,0}\right)$ for all the intervals are given, then the summation of two items is Δ. If there is more than one Δ < 0, we choose the smallest feasible one (Δ = −90 in the box in Table 3) to calculate current ${\bar{T}}_{min}$ for fixed *K*, as it also has the greatest influence on decreasing the total cost $C_{p}^{\prime }$. Hence, we can easily deduce the following Remark 1.

**Table 2:** Discount data of the two items.

Item *i*	Discount intervals	Price
2	0 ≤ *Q*_2_ < 500	3.25$
	500 ≤ *Q*_2_ < 1,000	3.20$
	1000 ≤ *Q*_2_ < 2,000	3.15$
5	*Q*_2_ ≥ 2,000	3.10$
	0 ≤ *Q*_5_ < 300	3.25$
	*Q*_5_ ≥ 300	3.20$

**Table 3:** Computational results for Δ.

Item 5		Item 2			
		*j* = 1	*j* = 2	*j* = 3	*j* = 4
	Δ_*i*⋅_	0	-25	-75	-175
*j* = 1	0	Δ = 0	Δ = -25	Δ = -75	Δ = -175
*j* = 2	**-15**	Δ = -15	Δ = -40	$\begin{array}{c} \Delta =-90 \end{array}$	Δ = -190
$S+\sum_{i=1}^{n}\left(s_{i}/k_{i}\right)$	141				
$min\{\left(S+\sum_{i=1}^{n}\left(s_{i}/k_{i}\right)+\Delta \right)\geq 0\}$	141 − 90 = 51 > 0				

‘*j*’ denotes *j*-th interval, *S* = 50, *s*_2_ = 46, *s*_3_ = 45, *K* = [1, 1, 1, 1, 1, 1].

**Remark 1**: For a given set of *k*_*i*_s, if *J*_*i*_ = 1, the optimal ${\bar{T}}^{*}$ reduces to T*, otherwise, if *j* are ascertained and $S+\sum_{i=1}^{n}\left(s_{i}/k_{i}\right)+\Delta \geq 0$, we can obtain that ${\bar{T}}^{*}$ is the optimal and less than or equal to T* (${\bar{T}}_{min}\leq {\bar{T}}^{*}\leq T^{*}$).

Remark 1 reveals that, the domain of optimal ${\bar{T}}^{*}$ is in [${\bar{T}}_{min},T^{*}$], that is to say, if the item is purchased at its non-discount price, the optimal ${\bar{T}}^{*}$ equals to T*, otherwise, ${\bar{T}}^{*}$ is calculated based on Eq (17). While the only difference between Eqs (6) and (17) lies in Δ. Therefore, the value of the optimal ${\bar{T}}^{*}$ is either calculated based on Eq (6), or obtained at the each threshold of discount interval, which is then applied to find the current best *k*_*i*_.

The next problem is to find a proper ${\bar{k}}_{i}$ that meets the above conditions. By referring to the basic constraint on the ordering quantity for interval *j* (*j* ≥ 1), we have *μ*_*i*,*j*−1_ ≤ *Q*_*i*_ < *μ*_*i*,*j*_, where *μ*_*i*,*j*−1_ and *μ*_*i*,*j*_ are the breakpoints. As $Q_{i}=D_{i}{\bar{k}}_{i}{\bar{T}}^{*}$, then, we have $\mu_{i,j-1}\leq D_{i}{\bar{k}}_{i}{\bar{T}}^{*}<\mu_{i,j}$, where $\frac{\mu_{i,j-1}}{D_{i}{\bar{T}}^{*}}\leq {\bar{k}}_{i}<\frac{\mu_{i,j}}{D_{i}{\bar{T}}^{*}}$. An integer real number ${\bar{k}}_{i}$ can be obtained in $\left[\left\lceil \frac{\mu_{i,j-1}}{D_{i}{\bar{T}}^{*}}\right\rceil ,\left\lfloor \frac{\mu_{i,j}}{D_{i}{\bar{T}}^{*}}\right\rfloor \right)$ for item *i* in the *j*-th discount interval. Moreover, if there have more than one feasible *k*_*i*_s in the domain, we choose the one that minimizes the current total cost *TC*.

Based on the remark, a simple iterative heuristic algorithm for *JRP* with multiple discounts is presented in Section 3.3.

### 3.3 An iterative heuristic algorithm

Numerous scholars development algorithms so solve JRP [39], such as *Power of Two* [40], spread-sheet technique [41], and Silver’s heuristic [37] and its extensions [42–44]. Since the heuristics are always problem pertinent, and the trivia in solving the discounted model is apparent, even to the models with only one type of discount scheme, the solving algorithms are complicated [16, 28], not to say multiple discount schemes are considered simultaneously. Therefore, to solve the proposed model, a heuristic algorithm is developed to deal with these multiple discounts. To the simple *JRP*, an iterative method was presented by Goyal [4] to find the optimal *T* and *k*_*i*_s, based on which the proposed algorithm is constructed. However, comparing to *T*, the optimal $\bar{T}$ is also interfered by Δ, so the most intricate part goes to find a best Δ. The simple case in Table 2 only provides us a rough sketch for computing Δ, as the number of items increases to 3 or more, it is not so easy to obtain Δ. Therefore, the following procedure provides a quick solution to find Δ*, see Algorithm 1.

**Algorithm 1** The procedure for obtaining ${\bar{T}}^{*}$

1: Compute $\nabla =S+\sum_{i=1}^{n}\left(s_{i}/k_{i}\right)$ and set a very large positive number *M*.

2: **for** *i* = 1 to *n*_2_ **do**

3:  **for** *j* = 1 to *J*_*i*_ **do**

4:   Compute and output Δ_*i*,*j*_.

5:  **end for**

6: **end for**

7: Formulate vector *V*_*i*_ as *V*_*i*_ = [Δ_*i*,1_, Δ_*i*,2_, … Δ_*i*,*J*_*i*__] and define set *DM* to contain all *V*s, *DM*(*i*) = *V*_*i*_.

8: Open spaces *P* for positioning the candidate Δ_*i*,*j*_ in *V*_*i*_, and *val* for containing the value of the candidate Δ_*i*,*j*_.

9: **for** *run* = 1 to Max_run **do**

10:  **for** *i* = 1 to *n*_2_ **do**

11:   [*val*(*i*), *P*(*i*)] = *min*(*DM*{*i*}(1 to *J*_*i*_)).

12:  **end for**

13:  Compute *Delta*, and *Delta* = *sum*(*val*).

14:  *temp* = ∇ + *Delta*.

15:  **if** *temp* ≤ 0 **then**

16:   // Select the minimum element in *val*, then position it in *DM*.

17:   [*result*, *index*] = *min*(*val*).

18:   *result* = *result* + *M*.

19:   *DM*{*index*}(*P*(*index*)) = *result*.

20:  **else**

21:   intermediateV(run) = temp;

22:   current best *Delta*: *CurBesDel* = *Delta*.

23:  **end if**

24: **end for**

25: best_intermediate_v = min(intermediateV), output the best found *Delta* as *BesFouDel* = *CurBesDel*, and corresponding position of discount interval of each item.

26: Compute the current best *T* based on Eq (17).

In Algorithm 1, a *nabla* symbol ∇ is applied to denote ‘$S+\sum_{i=1}^{n}\left(s_{i}/k_{i}\right)$’ and a very large number *M* is given for eliminating illegal numbers in line 1. Lines 2-6 is to calculate and output Δ_*ij*_, the result of which is then sent to a vector *V*_*i*_ in line 7, and a *cell array* *DM* is generated to contain all *V*_*i*_s. In lines 8-12, vectors *val* and *P* are defined as two arrays to contain the minimum value and the corresponding position of Δ_*ij*_, respectively. Lines 9-24 are presented to illustrate the procedure for obtaining all feasible values of ∇ + Δ, which is contained in an intermediate vector *intermediateV*. The minimized element in *intermediateV* is output in line 25 and which is then to applied to compute *T* in line 26.

Based on the obtained $\bar{T}$, Algorithm 2 is provided to update *k*_*i*_s.

**Algorithm 2** The procedure for updating *k*_*i*_

1: Set ‘*n*’ as the total number of items, and predefine vectors *MinK* and *MaxK* to contain the smallest and largest *k*_*i*_ of item *i*, and initially, *MinK* = *MaxK* = *ones*(1, *n*).

2: **for** *i* = 1 to *n* **do**

3:  **if** item $i\notin N_{2}$ **then**

4:   Compute *k*_*i*_ based on Eq (7).

5:  **else**

6:   //Compute the lower and upper bounds of *k*_*i*_ according to $\left\lceil \frac{\mu_{i,j-1}}{D_{i}{\bar{T}}^{*}}\right\rceil \leq k_{i}<\left\lfloor \frac{\mu_{i,j}}{D_{i}{\bar{T}}^{*}}\right\rfloor$ and the thresholds of *j*-th interval of item *i*, and

7:   $MinK\left(i\right)=\left\lceil \frac{\mu_{i,j-1}}{D_{i}{\bar{T}}^{*}}\right\rceil$ and $MaxK\left(i\right)=\left\lfloor \frac{\mu_{i,j}}{D_{i}{\bar{T}}^{*}}\right\rfloor$.

8:   **for** *k* = *MinK*(*i*) to *MaxK*(*i*) − 1 **do**

9:    Compute and output the minimum *TC*.

10:   **end for**

11:   Output the best current best *k*_*i*_ according to the minimum *TC*.

12:  **end if**

13: **end for**

14: Output the current best *K*.

Algorithm 2 offers two main ways for computing the *k*_*i*_s, for items purchased under ND, AD and BD, the *k*_*i*_s are obtained by Eq (7), the procedure of which is provided in lines 3-4, for items purchased under ID, a new *k*_*i*_ is obtained through the interval thresholds and updated by finding a smaller total cost, the procedure of which is provided in lines 5-11. Then, the current best *K* is output.

The synthetical procedure for solving the proposed problem is presented in Algorithm 3, the pseudocodes of which are given as follows. The first three lines of Algorithm 3 are preparation procedures for initializing the input parameters, such as *K*, *TC*, and the maximum accumulative computation times as *X*. The counter is initialized as ‘*counter* = 0’ in line 3. The loop in lines 5-15 depicts the main procedure for updating *T* (by calling Algorithm 1) and *K* (by calling Algorithm 2) when and only when the new total cost is smaller than current best total cost. Lines 16-20 present the loop for running the counter and when the maximum accumulative computation times reached to *X*, Algorithm 3 is terminated.

**Algorithm 3** The synthetical procedure for the proposed model

1: Initialize *K* as a unit vector, assign *TC* a very large number and set it as ‘*CurBesTC*’.

2: Set accumulative computation times as *X* and ‘*count*’ as the counter.

3: *count* = 0.

4: **for** *G* = 1 to *Max*_*G* **do**

5:  Call Algorithm 1 for computing a *newT* and output the position of *j*-th interval of item *i*.

6:  Call Algorithm 2 for updating *k*_*i*_, output the local best found *K* as *newK* and the corresponding minimum *TC* as the new *newTC*.

7:  **if** *newTC* < *CurBesTC* **then**

8:   *T* = *newT*. // *T* is updated

9:   *K* = *newK*. // *K* is updated

10:   *CurBesTC* = *newTC*. // *TC* is updated

11:   *count* = 0. // recount

12:  **else**

13:   Keep *T*(*G*), *K*(*G*) and *CurBesTC* unchanged.

14:   ++*count*.

15:  **end if**

16:  **if** *count* == *X* and *CurBesTC* is unchanged **then**

17:   Break. // Break out of the loop

18:  **else**

19:   Continue. // Continue the loop

20:  **end if**

21: **end for**

**Fig 2:**
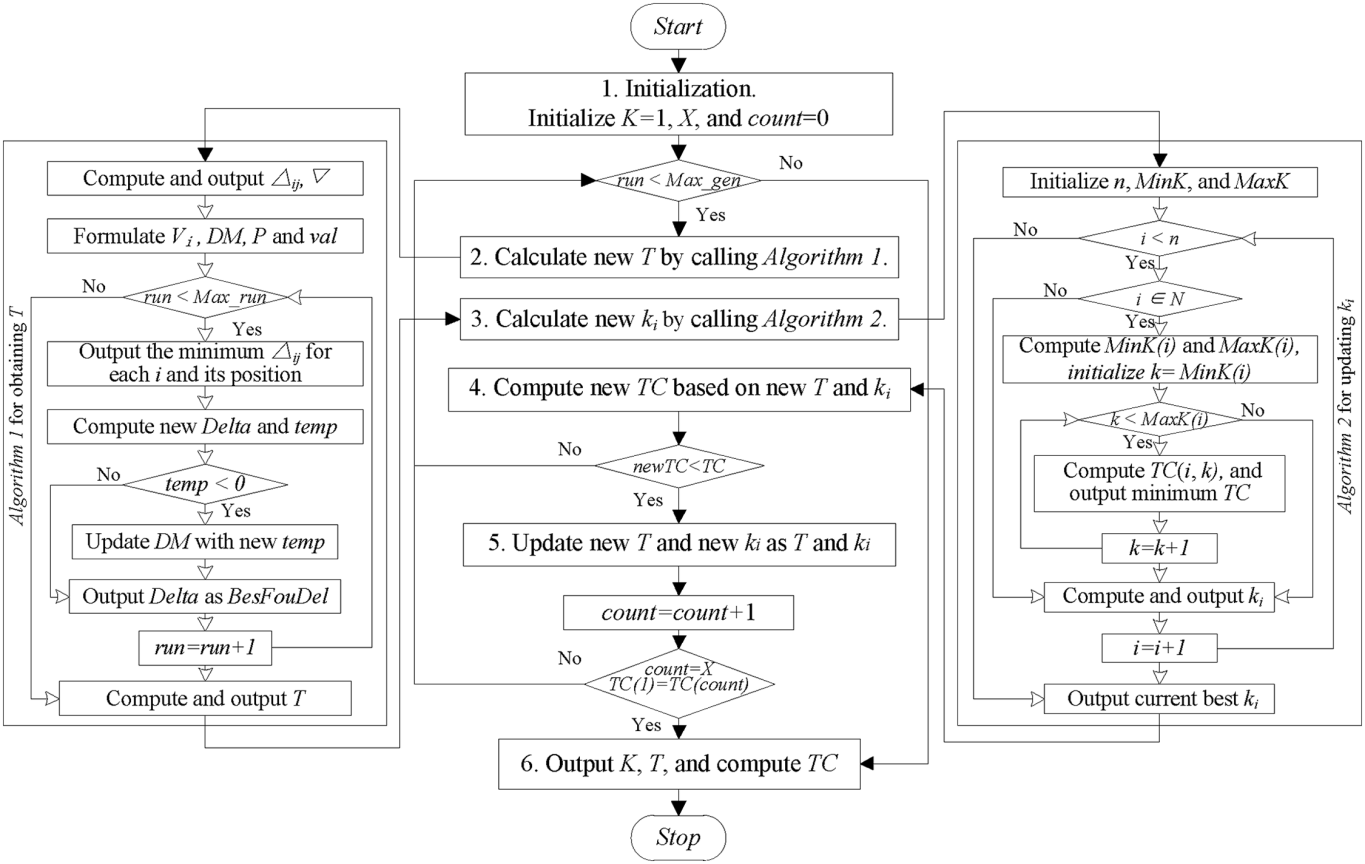
Flow chart of the algorithm.

The flow chart of the synthetical heuristic algorithm is given as Fig 2. In Fig 2, the mini-figure in the middle is to illustrate the 6 searching steps of Algorithm 3. The left mini-figure is to call Algorithm 1 to calculate the current-best *T*, see line 5 of Algorithm 3. The right mini-figure is to call Algorithm 2 to update *k*_*i*_ based on the returned current-best *T*. After certain steps of iteration, the final result is output as our best-found result.

## 4 Numerical experiment

In this section, a *JRP* case with 6 items is presented to demonstrate the constructed model and the heuristic algorithm. In the case, a supplier supplies multi-item to a single B2C company. In order to promote the sales of these items, the supplier offers different promoting discount schemes. The basic data for the case is presented in Table 4 and the data on quantity discounts are presented in Table 5.

**Table 4:** The data for the *JRP* case.

Item *i*	1	2	3	4	5	6
*D*_*i*_	10,000	5,000	3,000	1,000	600	200
*h*_*i*_	1	1	1	1	1	1
*s*_*i*_	45	46	47	44	45	47
S	100	100	100	100	100	100
*c*_*i*_	0.10	0.10	0.10	0.10	0.10	0.10

**Table 5:** Discount schedule.

Item *i*	Discount types	Schedule	Price
1	AD	0 ≤ *Q*_1_ < 500	0.10$
		500 ≤ *Q*_1_ < 1,000	0.09$
		1000 ≤ *Q*_1_ < 2,000	0.08$
		*Q*_1_ ≥ 2,000	0.07$
2	ID	0 ≤ *Q*_2_ < 500	0.10$
		500 ≤ *Q*_2_ < 1,000	0.09$
		1000 ≤ *Q*_2_ < 2,000	0.08$
		*Q*_2_ ≥ 2,000	0.07$
3	AD	0 ≤ *Q*_3_ < 500	0.10$
		500 ≤ *Q*_3_ < 1,000	0.09$
		*Q*_3_ ≥ 1,000	0.08$
4	ND	—	0.10$
5	ID	0 ≤ *Q*_5_ < 300	0.10$
		*Q*_5_ ≥ 300	0.09$
6	BD	0 ≤ *C*_*BD*_ < 10$	0.00%
		10$≤*C*_*BD*_	10%

Specifically, from Table 5 we can observe that *n*_0_ = 1, *n*_1_ = 2, *n*_2_ = 2, *n*_3_ = 1, the proposed algorithm is applied to solve the case, and if all items are purchased without any discount considerations, the solving algorithm is reduced to solve *JRP* with ND. To make clearly comparisons, we assume all the items are purchased with all-unit quantity discount (AD) considering the same purchasing cost structure as that in Table 5 and quantity structure as that in Table 4. The comparison results of *JRP* with (all items are presumed to be purchased under) *JRP* with (all items are presumed to be purchased under) AD, *JRP* with (all items are presumed under) ID, *JRP* with (all items are presumed to be purchased under) BD and *JRP* with multiple discounts (MD) using the proposed heuristic are presented in Tables 6 and 7.

**Table 6:** Comparisons of *JRP* with different discounts.

	*K*	*T*	TC
*JRP* with ND	1,1,1,1,1,4	0.1822	8,337.86
*JRP* with AD	1,1,1,1,1,4	0.1822	8,049,86
*JRP* with ID	1,1,1,1,2,2	0.1523	8,555,01
*JRP* with BD	1,1,1,1,1,4	0.1822	8,049,96
*JRP* with MD	1,1,1,1,2,4	0.1628	8,204,21

**Table 7:** The schedule information under different discount schemes and Q*.

Item	ND		AD and ID				BD			MD			
	Schedule	Q*	Schedule	Price	$Q_{1}^{*}$	$Q_{2}^{*}$	Schedule	Price	Q*	Types	Schedule	Price	Q*
1	0 ≤ *Q*_1_ < 10000	1822	0 ≤ *Q*_1_ < 500	0.10$			0 ≤ *Q*_1_ < 500	0%		AD	0 ≤ *Q*_1_ < 500	0.10$	
			500 ≤ *Q*_1_ < 1,000	0.09$			500 ≤ *Q*_1_ < 1,000	10%			500 ≤ *Q*_1_ < 1,000	0.09$	
			1000 ≤ *Q*_1_ < 2,000	0.08$	1822	1523	1000 ≤ *Q*_1_ < 2,000	20%	1822		1000 ≤ *Q*_1_ < 2,000	0.08$	1628
			*Q*_1_ ≥ 2,000	0.07$			*Q*_1_ ≥ 2,000	30%			*Q*_1_ ≥ 2,000	0.07$	
2	0 ≤ *Q*_2_ < 5000	911	0 ≤ *Q*_2_ < 500	0.10$			0 ≤ *Q*_2_ < 500	0%		ID	0 ≤ *Q*_2_ < 500	0.10$	
			500 ≤ *Q*_2_ < 1,000	0.09$	911	762	500 ≤ *Q*_2_ < 1,000	10%	911		500 ≤ *Q*_2_ < 1,000	0.09$	814
			1000 ≤ *Q*_2_ < 2,000	0.08$			1000 ≤ *Q*_2_ < 2,000	20%			1000 ≤ *Q*_2_ < 2,000	0.08$	
			*Q*_2_ ≥ 2,000	0.07$			*Q*_2_ ≥ 2,000	30%			*Q*_2_ ≥ 2,000	0.07$	
3	0 ≤ *Q*_3_ < 3000	547	0 < ≤*Q*_3_ < 500	0.10$		457	0 < ≤*Q*_3_ < 500	0%		AD	0 < ≤*Q*_3_ < 500	0.10$	488
			500 ≤ *Q*_3_ < 1,000	0.09$	547		500 ≤ *Q*_3_ < 1,000	10%	547		500 ≤ *Q*_3_ < 1,000	0.09$	
			*Q*_3_ ≥ 1,000	0.08$			*Q*_3_ ≥ 1,000	20%			*Q*_3_ ≥ 1,000	0.08$	
4	0 ≤ *Q*_4_ < 1000	182	0 ≤ *Q*_4_ < 1000	0.10$	182	152	0 ≤ *Q*_4_ < 1000	0%	182	ND	0 ≤ *Q*_4_ < 1000	0.10$	163
5	0 ≤ *Q*_5_ < 600	109	0 ≤ *Q*_5_ < 300	0.10$	109	183	0 ≤ *Q*_5_ < 300	0%	109	ID	0 ≤ *Q*_5_ < 300	0.10$	195
			*Q*_5_ ≥ 300	0.09$			*Q*_5_ ≥ 300	10%			*Q*_5_ ≥ 300	0.09$	
6	0 ≤ *Q*_6_ < 200	146	0 ≤ *Q*_6_ < 10$	0.00%		61	0 ≤ *Q*_6_ < 10$	0%		BD	0 ≤ *Q*_6_ < 10$	0.00%	
			*Q*_6_ ≥ 10$	10.0%	146		*Q*_6_ ≥ 10$	10%	146		*Q*_6_ ≥ 10$	10.0%	130

$Q_{1}^{*}$: Q* under AD, $Q_{2}^{*}$: Q* under ID.

The results in Tables 6 and 7 tell that,

(1) Taking the basic cycle time and replenishment frequencies for discussion. The basic cycle time *T* under different discount schemes shows different features, *T*s and *K*s of *JRP* with ND, with AD and with BD are the same, that is because all these *T*s and *K*s are obtained by Eq (7), but *T*s of *JRP* with ID and MD are shortened as the value of *T* is interfered ∇ and Δ, the replenishment frequencies of *JRP* with ID and *JRP* with MD are interfered by the obtained *T*, correspondingly.

(2) Taking the total cost for discussion, the results of *TC* reveal the roles and magnitudes of different schemes on *TC*. From the perspective of the supplier, the best discount offer for him/her is to adopt the incremental discount scheme, as it can bring him/her more benefits. When standing at the side of the buyer, the best offer is definitely the *JRP* with AD or with BD, as he/she can more cost decreasing than *JRP* with ND and with ID. However, the suppliers and buyers who want to build a long term stable supply chain, the MD scheme may be the most promising scheme form them. MD scheme plays mediate intermediate role comparing to *JRP* with AD (with BD) and *JRP* with ID, also *TC* under MD is smaller than that under ND in above case.

(3) On how different discount schemes impact the order quantity per order, Table 7 gives some hints. To the items ordered under ND, AD, BD, the role of introduction of discount mainly reflects in decreasing the total cost. To the items ordered under ID and MD, respectively, the role of introduction the discount reflects both in decreasing the total cost of *JRP* and order quantities of relevant items. Also, our model testified the assumption of [28] that the all-unit quantity discount and total business volume discount may have the similar effects if the order quantity is specific and can be counted.

## 5 Conclusions

In this paper, we provide a new focus on *JRP* with multiple discount schemes. By referring to the work of predecessors on supplier selection and multi-item replenishment considering different discount types, a new *JRP* model is constructed considering three discount types simultaneously to investigate the joint effects of discount schemes on the decisions of replenishment cycle time and frequencies of each item. In light of the *NP-hard* nature of *JRP*, a heuristic algorithm is presented to solve the proposed model. Through numerical experiments on different *JRP*s with different discount type combinations, we verify that both the supplier and the buyer would be benefited by formulating a multiple discount contract.

This research aims to give a new extension of *JRP* and a simple heuristic for solving the new model, but the performance of the proposed heuristic is not fully verified comparing to the existed evolutionary algorithms. Thus, in our following research, we would spare our energy in finding some more efficient and effective algorithms to solve the proposed model, and the other is to extend the current problem to *JRP*s with delivery consideration.
